# Decreased plasma ELABELA level as a novel screening indicator for heart failure: a cohort and observational study

**DOI:** 10.1038/s41598-024-61480-x

**Published:** 2024-05-17

**Authors:** Chunju Liu, Jianhua Xiong, Xiaoli Yi, Shanshan Song, Huiru Yang, Wenting Tan, Xiaojun Yang, Lixiang Zheng, Jun Yu, Chuanming Xu

**Affiliations:** 1 Translational Medicine Centre, Jiangxi University of Chinese Medicine, Nanchang, 330004 China; 2 Department of Clinical Laboratory, Affiliated Hospital of Jiangxi University of Chinese Medicine, Nanchang, 330006 China; 3 Department of Cardiology, Affiliated Hospital of Jiangxi University of Chinese Medicine, Nanchang, 330006 China; 4 College of Traditional Chinese Medicine, Jiangxi University of Chinese Medicine, Nanchang, 330004 China; 5 Center for Metabolic Disease Research and Department of Cardiovascular Sciences, Lewis Katz School of Medicine, Temple University, Philadelphia, PA 19140 USA

**Keywords:** ELABELA, Apelin, BNP, Heart failure, Diagnosis, Biomarkers, Heart failure

## Abstract

The predictive power of B-type natriuretic peptide (BNP) and left ventricular ejection fraction (LVEF) is limited by its low specificity in patients with heart failure (HF). Discovery of more novel biomarkers for HF better diagnosis is necessary and urgent. ELABELA, an early endogenous ligand for the G protein-coupled receptor APJ (Apelin peptide jejunum, Apelin receptor), exhibits cardioprotective actions. However, the relationship between plasma ELABELA and cardiac function in HF patients is unclear. To evaluate plasma ELABELA level and its diagnostic value in HF patients, a total of 335 patients with or without HF were recruited for our monocentric observational study. Plasma ELABELA and Apelin levels were detected by immunoassay in all patients. Spearman correlation analysis was used to analyze the correlation between plasma ELABELA or Apelin levels and study variables. The receiver operating characteristic curves were used to access the predictive power of plasma ELABELA or Apelin levels. Plasma ELABELA levels were lower, while plasma Apelin levels were higher in HF patients than in non-HF patients. Plasma ELABELA levels were gradually decreased with increasing New York Heart Association grade or decreasing LVEF. Plasma ELABELA levels were negatively correlated with BNP, left atrial diameter, left ventricular end-diastolic diameter, left ventricular end-systolic diameter, and left ventricular posterior wall thickness and positively correlated with LVEF in HF patients. In contrast, the correlation between plasma Apelin levels and these parameters is utterly opposite to ELABELA. The diagnostic value of ELABELA, Apelin, and LVEF for all HF patients was 0.835, 0.673, and 0.612; the sensitivity was 62.52, 66.20, and 32.97%; and the specificity was 95.92, 67.23, and 87.49%, respectively. All these parameters in HF patients with preserved ejection fraction were comparable to those in total HF patients. Overall, plasma ELABELA levels were significantly reduced and negatively correlated with cardiac function in HF patients. Decreased plasma ELABELA levels may function as a novel screening biomarker for HF. A combined assessment of BNP and ELABELA may be a good choice to increase the accuracy of the diagnosis of HF.

## Introduction

Heart failure (HF) is recognized as a major clinical and global public health concern that affected nearly 64.3 million people worldwide in 2017^1^. In particular, HF has been a leading cause of the high mortality and morbidity of cardiovascular diseases with an average annual mortality of 25–40% worldwide^2^. HF not only affects the elderly but also burdens the young population (< 50 years old) with a continuous rise in incidence^3,4^. It is vital to achieve early diagnosis and effective risk stratification to improve the management of HF patients. B-type natriuretic peptide (BNP) and left ventricular ejection fraction (LVEF) have been widely used in the diagnosis and prognosis of HF^5^. However, their predictive power was limited by their low specificity in clinical applications^6,7^. A combined assessment of BNP and other factors can improve HF diagnosis^8^. Thus, it is necessary and urgent to discover more novel biomarkers for HF diagnosis.

The Apelinergic system consists of a G protein-coupled receptor APJ (Apelin peptide jejunum, Apelin receptor, encoded by *Aplnr*)^9^ and two endogenous peptide ligands ELABELA (encoded by *Apela*, also called Toddler)^10,11^ and Apelin (encoded by *Apln*)^12^. The Apelinergic system significantly regulates cardiovascular homeostasis and functions as a potential therapeutic target of cardiovascular diseases^13,14^. Notably, the Apelin peptide could ameliorate acute HF by inhibiting endoplasmic reticulum stress^15^, ELABELA could protect against hypertensive-induced cardiac damage by inhibiting FoxM1/ACE signaling^16^ and improve left ventricular filling in cecal ligation puncture rats^17^. These results indicated the participation of Apelin and ELABELA in preventing HF. However, studies have indicated that ELABELA may be more efficient than Apelin^18^.

Accumulating evidence showed that Apelin and ELABELA exert similar important bioeffects, including cardiorenal protective action, anti-hypertension action, and positive inotropic effect^14,18,19^. As reported, the status of the plasma Apelin levels in HF patients is still controversial. Many studies have demonstrated decreased plasma Apelin levels^20–26^, while several other studies have indicated increased or unchanged plasma Apelin levels in HF patients^27–30^. Similarly, plasma ELABELA levels were markedly elevated in patients with myocardial infarction^31,32^ and complete atrioventricular block^33^ but reduced significantly in patients with congenital heart disease^34^ and atrial fibrillation^35,36^. Plasma ELABELA levels were also decreased in patients with hypertension^37^ and renal impairment^38^. Of note, hypertension and renal impairment are two independent risk factors for HF progression^39,40^, implying the potential biomarker function of ELABELA for HF. However, only one small cohort study indirectly demonstrated the correlation between plasma ELABELA and cardiac function in HF patients^41^. The levels of plasma ELABELA were significantly decreased in hypertensive patients with HF compared to those in hypertensive patients without HF^41^. In the present study, with the maximum exclusion of interference from other complications, we further evaluated the plasma ELABELA and Apelin levels and investigate the association between plasma ELABELA or Apelin levels and cardiac function in HF patients. Additionally, we also compared the diagnostic value of ELABELA, Apelin, and LVEF in HF patients.

## Methods

### Study population

The studies involving human participants were reviewed and approved by the Ethics Committee of the Affiliated Hospital of the Jiangxi University of Chinese Medicine (JZFYLL20230208002) and performed in accordance with the World Medical Association Declaration of Helsinki—Ethical Principles for Medical Research Involving Human Subjects. All individual patients/participants provided their written informed consent and clinical characteristics to participate in this study at enrollment. All subjects were recruited in the Department of Cardiology, Affiliated Hospital of Jiangxi University of Chinese Medicine, between December 2022 and July 2023 and divided into Non-HF and HF groups. All the laboratory assessments, except plasma ELABELA and Apelin levels, were conducted in the clinical laboratory center according to the standard protocols. Inclusion criteria for diagnosis and classification of HF, including (1) typical signs (i.e., dyspnea), (2) typical symptoms (i.e., pulmonary rales), (3) increased plasma brain natriuretic peptide (BNP) concentrations [> 95 pg/ml according to the New York Heart Association (NYHA) functional classification.], (4) ultrasound cardiogram report (impaired cardiac function evaluated by echocardiography), and (5) X-ray examination (i.e., enlarged heart shadow) were based on the 2021 ESC Guidelines for the diagnosis and treatment of HF^5^. Heart failure with preserved EF (HFpEF), mid-range EF (HFmrEF), and reduced ejection fraction (HFrEF) was defined as EF ≥ 50%, > 40 but < 50, and ≤ 40%, respectively^5^. The exclusion criteria were: (1) heart diseases (i.e., atrial fibrillation, congenital heart disease, acute myocardial infarction, cardiomyopathy), (2) severe renal dysfunction (grade 4 or higher), (3) malignant tumor, (4) inferior airway diseases (i.e., acute pulmonary embolism, severe disease of lung parenchyma), (5) autoimmune related diseases (i.e., severe infection, autoimmune disease, autoimmune deficiency disease), and (6) acute stroke. In this regard, on the premise of following the inclusion and exclusion criteria, patients from the Department of Cardiology with plasma BNP concentrations ≥ 95 pg/ml were included in the HF group and patients without symptoms and signs of HF with plasma BNP concentrations < 95 pg/ml were included in the Non-HF group. All of the patients/participants did not receive the optimized treatment before collecting blood samples.

### ELISA assays for plasma ELABELA and Apelin

All the fasting blood samples were collected from a peripheral vein of all patients within 24 h of admission. Upon collection, blood samples were immediately centrifuged for 5 min at 4 °C and 3000 rpm to separate plasma. The plasma samples were stored at − 80 °C for ELABELA and Apelin analysis by ELISA using the commercialized human ELABELA ELISA Kit (S-1508, Peninsula Laboratories International, Inc. USA) and Apelin ELISA kit (E01T0015, Bluegene Tech Inc., Shanghai, China) according to the manufacturers’ instructions, respectively. For the measurement of plasma ELABELA, the plasma samples were appropriately extracted.

### Statistical analysis

Continuous data were expressed as mean ± standard deviation (SD) for normally distributed data, median and interquartile range (IQR) for non-normally distributed data, and categorical variables as number and percentage. Student’s t-test was used for intergroup differences in continuous normally distributed variables between two groups, one-way analysis of variance followed by Bonferroni comparisons and unpaired tests was used for continuous normally distributed variables among more than two groups. Spearman correlation analysis was used to correlate plasma ELABELA or Apelin levels and study variables. The clinical characteristics associated with HF were analyzed through univariate and multivariate logistic regression in all subjects. The diagnostic value of plasma ELABELA, Apelin, and LVEF was assessed by determining the area under the receiver operating characteristic (ROC) curves (AUC) using the DeLong test. All tests were two-sided, and *P* < 0.05 was considered statistically significant. The statistical analyses were performed using MedCalc Version 22.017 (MedCalc Software Ltd, Ostend, Belgium).

### Ethics approval

The studies involving human participants were reviewed and approved by the Affiliated Hospital of the Jiangxi University of Chinese Medicine (JZFYLL20230208002). The patients/participants provided written informed consent to participate in this study.

### New and noteworthy

The predictive power of BNP and LVEF is limited by its low specificity in patients with HF. We reported that plasma ELABELA was significantly reduced and negatively correlated with cardiac function in HF patients, utterly opposite to the changes in plasma BNP and Apelin. Plasma ELABELA might be superior to Apelin and LVEF for the diagnosis and prognosis of HF, at least in patients with HFpEF. Combined assessment of BNP and ELABELA may provide potential benefits for the diagnosis of HF.

## Results

### The baseline characteristics of patients

A total of 335 participants with and without HF were enrolled in the Non-HF group (n = 119, 68.4 ± 12.2 years) and the HF group (n = 216, 69.6 ± 11.6 years, *p* = 0.176 vs. Non-HF group), respectively; the other baseline clinical characteristics are shown in Table 1. There were no significant differences in sex, body mass index (BMI), comorbidities, or blood pressure between the two groups. For complications, the incidence of coronary heart disease was higher in the HF than in the Non-HF group (22.2 vs. 16.8%, *p* = 0.047). Data from laboratory examinations revealed that plasma BNP, creatinine, urea nitrogen, uric acid, and low-density lipoprotein cholesterol (LDL-c) levels were all higher in the HF group compared to the non-HF group (*P* < 0.05). In contrast, the plasma total cholesterol and triglyceride levels were lower in the HF group compared to the non-HF group (*P* < 0.05). Compared to the non-HF group, the HF group showed lower left ventricular ejection fraction (LVEF) and higher left atrial diameter (LAD), left ventricular end-diastolic diameter (LVEDd), left ventricular end-systolic diameter (LVEDs), and left ventricular posterior wall (LVPW) thickness (*p* < 0.05), with no significant difference in interventricular septum thickness (IVST) and right ventricular internal dimension diameter (RVIDd) between non-HF and HF groups.

**Table 1:** Baseline characteristics and laboratory data of heart failure patients.

Parameters	Total (n = 335)	Non-HF (n = 119)	HF (n = 216)	*P* value (Non-HF vs. HF)
Age, years	69.5 ± 13.0	68.4 ± 12.2	69.6 ± 11.6	0.176
Male	169/335 (50.5%)	61/119 (51.3%)	108/216 (50.0%)	1.000
Body mass index, kg/m^2^	26.2 ± 2.5	25.9 ± 3.2	26.3 ± 3.3	0.345
Comorbidities				
Coronary artery disease	68/335 (20.3%)	20/119 (16.8%)	48/216 (22.2%)	0.047*
Diabetes mellitus	5/335 (1.5%)	0/119 (0.0%)	5/216 (2.3%)	0.058
Chronic renal failure	3/335 (0.9%)	0/119 (0.0%)	3/216 (1.4%)	0.062
Hypertension	60/335 (17.9%)	22/119 (18.5%)	38/216 (17.6%)	0.453
Systolic blood pressure, mmHg	128.4 ± 15.0	128.0 ± 12.3	128.6 ± 16.4	0.744
Diastolic blood pressure, mmHg	76.2 ± 9.6	77.3 ± 8.9	75.6 ± 10.0	0.120
Mean arterial pressure, mmHg	93.6 ± 10.8	94.2 ± 9.5	93.2 ± 11.5	0.439
Heart rate, bpm	76.8 ± 8.1	75.8 ± 7.6	77.4 ± 8.3	0.080
Laboratory data				
Plasma BNP, pg/ml	1172.5 (65.4, 1100.4)	55.1 (38.7, 69.4)	1788.2 (199.1, 2160.2)	< 0.001***
Plasma creatine, μmol/l	79.5 (55.6, 78.4)	73.4 (50.8, 90.6)	88.4 (57.5, 88.6)	0.251
Plasma urea nitrogen, μmol/l	7.3 (4.8, 7.6)	5.8 (4.7, 6.7)	8.2 (5.0, 8.2)	< 0.001***
Plasma uric acid, μmol/l	353.9 (279.0, 416.0)	337.4 (264.0, 390.0)	363.0 (288.0, 426.8)	0.039*
Plasma LDL-c, mmol/l	2.4 ± 1.0	2.3 ± 0.9	2.6 ± 1.0	0.005**
Plasma HDL-c, mmol/l	1.4 ± 0.4	1.4 ± 0.3	1.3 ± 0.4	0.123
Plasma total cholesterol, mmol/l	4.4 ± 1.2	4.7 ± 1.1	4.2 ± 1.2	< 0.001***
Plasma triglyceride, mmol/l	1.4 ± 1.0	1.6 ± 1.2	1.3 ± 0.8	0.018*
Echocardiographic data				
LVEF, %	61.8 ± 7.6	64.3 ± 4.3	60.4 ± 8.7	< 0.001***
LAD, mm	34.8 ± 6.5	32.2 ± 3.7	36.2 ± 7.2	< 0.001***
LVEDd, mm	46.0 ± 6.1	44.3 ± 3.8	47.0 ± 6.9	< 0.001***
LVEDs, mm	30.6 ± 5.8	28.9 ± 3.1	31.5 ± 6.7	< 0.001***
IVST, mm	9.8 ± 1.1	9.7 ± 1.0	9.9 ± 1.1	0.101
LVPW, mm	9.5 ± 1.2	9.3 ± 1.0	9.6 ± 1.3	0.020*
RVIDd, mm	20.3 ± 2.5	20.0 ± 1.9	20.5 ± 2.8	0.062
NYHA function grade				
NYHA I	57/335 (17.0%)	–	57/216 (26.4%)	–
NYHA II	32/335 (9.6%)	–	32/216 (14.8%)	–
NYHA III	39/335 (11.6%)	–	39/216 (18.1%)	–
NYHA IV	88/335 (26.3%)	–	88/216 (40.7%)	–

BNP, B-type natriuretic peptide; LDL-c, low-density lipoprotein cholesterol; HDL-c, high-density lipoprotein cholesterol; LAD, left atrial diameter; LVEDd, left ventricular end-diastolic diameter; LVEDs, left ventricular end-systolic diameter; LVEF, left ventricular ejection fraction; IVST, interventricular septum thickness; LVPW, left ventricular posterior wall thickness; RVIDd, right ventricular internal dimension diameter. HF, heart failure. NYHA, New York Heart Association. *P < 0.05; **P < 0.01; ***P < 0.001.

Significant values are in bold.

### Plasma BNP levels in patients with HF and without HF

Plasma BNP levels were higher in HF patients than in non-HF groups (Table 1). We further divided the 216 HF patients into four subgroups [NYHA I (57/216), II (32/216), III (39/216), and IV (88/216)] according to the classification of NYHA, or HFpEF (191/216), HFmrEF (15/216), and HFrEF (10/216) groups determined by LVEF. Plasma BNP levels were further increased with severity stratified by NYHA grade (Table 2) and the continuous reduction of LVEF (Table 3), and a similar gradient reduction of LVEF was observed in patients in both classification models.

**Table 2:** Plasma levels of B-type natriuretic peptide (BNP), ELABELA, Apelin, and left ventricular ejection fraction (LVEF) for heart failure (HF) patients by New York Heart Association (NYHA) grade.

Parameters	Non-HF (n = 119)	HF (n = 216)			
		NYHA I (n = 57)	NYHA II (n = 32)	NYHA III (n = 39)	NYHA IV (n = 88)
Plasma BNP, pg/ml	55.1 ± 19.2	142.3 ± 35.8***	327.7 ± 72.5***^###^	684.0 ± 164.2***^###$$$^	3874.7 ± 649.7***^###$$$&&&^
Plasma ELABELA, ng/ml	13.7 ± 3.0	10.7 ± 2.9*	9.2 ± 2.5***^##^	7.2 ± 3.4***^###$^	5.5 ± 3.6***^###$$$&&^
Plasma Apelin, pg/ml	51.9 ± 19.6	90.7 ± 15.1	209.4 ± 34.5***^#^	161.8 ± 25.3***^##^	181.3 ± 38.7***^#^
LVEF, %	64.3 ± 4.3	63.5 ± 6.2	62.7 ± 7.3	59.9 ± 8.4***^#^	58.1 ± 9.9***^###$^

*P < 0.05 and ***P < 0.001 *vs*. Non-HF; ^#^*P* < 0.05, ^##^*P* < 0.01, and ^###^*P* < 0.001 *vs*. NYHA I; ^$^*P* < 0.05 and ^$$$^*P* < 0.001 *vs*. NYHA II; ^&&^*P* < 0.01 and ^&&&^*P* < 0.001 *vs*. NYHA III.

**Table 3:** Plasma levels of B-type natriuretic peptide (BNP), ELABELA, Apelin, and left ventricular ejection fraction (LVEF) in patients with HFpEF, HFmrEF, and HFrEF.

Parameters	Non-HF (n = 119)	HF (n = 216)	HF (n = 216)		
			HFpEF (n = 191)	HFmrEF (n = 15)	HFrEF (n = 10)
Plasma BNP, pg/ml	55.1 ± 19.2	1788.2 ± 904.3***	1523.9 ± 218.9***	3795.5 ± 699.9***^##^	3826.0 ± 792.4***^##^
Plasma ELABELA, ng/ml	13.7 ± 3.0	8.4 ± 4.5***	8.8 ± 4.5***	6.3 ± 4.7***^#^	4.6 ± 3.0***^##$^
Plasma Apelin, pg/ml	51.9 ± 19.6	152.2 ± 68.9***	148.2 ± 25.6***	190.0 ± 27.7***	166.8 ± 11.0***
LVEF, %	64.3 ± 4.3	60.5 ± 8.7	63.1 ± 4.8	44.9 ± 2.6***^###^	35.1 ± 3.4***^###$$$^

HEpEF, heart failure with preserved ejection fraction; HFmrEF, heart failure with mid-range ejection fraction; HFrEF, Heart failure with reduced ejection fraction. ***P < 0.001 *vs*. Non-HF; ^#^*P* < 0.05, ^##^*P* < 0.01, and ^###^*P* < 0.001 *vs*. HEpEF; ^$^*P* < 0.05 and ^$$$^*P* < 0.001 *vs*. HFmrEF.

### Plasma ELABELA levels in patients with HF and without HF

**Figure 1:**
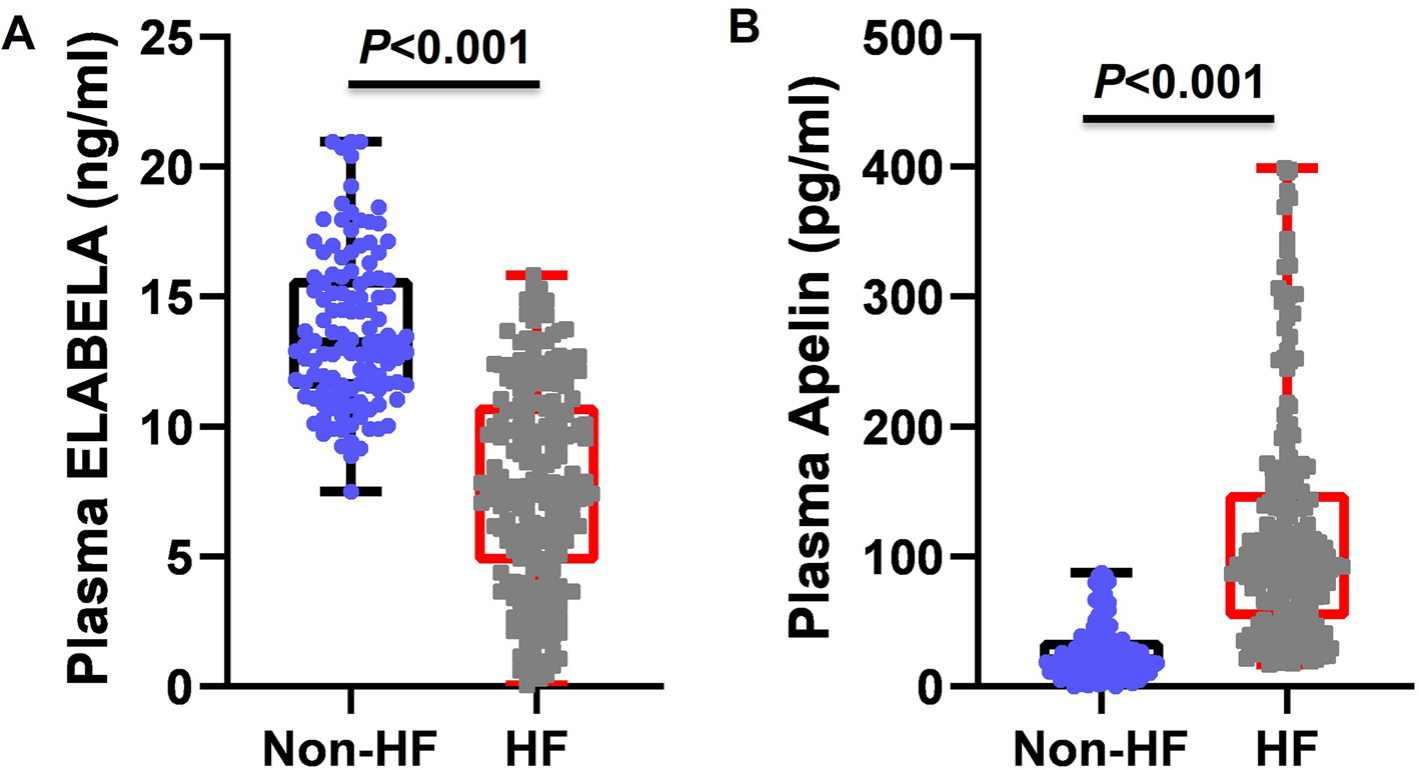
Plasma ELABELA (**A**) and Apelin (**B**) levels in patients with heart failure.

The levels of plasma ELABELA in HF patients were significantly lower than those in non-HF groups (7.3 ± 3.9 vs. 13.7 ± 3.0 ng/ml, p < 0.001, Fig. 1A). They were further significantly reduced with severity stratified by NYHA grade (NYHA I 12.3 ± 3.5 *vs*. NYHA II 9.9 ± 2.8 *vs*. NYHA III 7.7 ± 4.0 *vs*. NYHA IV 5.6 ± 3.9 ng/ml) (Table 2). Interestingly, the mean plasma ELABELA levels of the HFpEF, HFmrEF, and HFrEF groups were significantly lower than in the non-HF group. The plasma ELABELA levels in HFpEF (8.8 ± 4.5 ng/ml), HFmrEF (6.3 ± 4.7 ng/ml), and HFrEF (4.6 ± 3.0 ng/ml) groups were decreased sequentially with statistical significance (Table 3).

### Plasma Apelin levels in patients with HF and without HF

Plasma Apelin levels were higher in HF patients than in non-HF groups (116.5 ± 16.8 vs. 27.2 ± 11.3 ng/ml, p < 0.001, Fig. 1B) and in HF patients with NYHA II, III, and IV grade than in non-HF groups with no significant difference between non-HF and NYHA I patients (Table 2). The severity stratified by NYHA grade (Table 2) or LVEF levels (Table 3) did not affect the plasma levels of Apelin in HF patients.

### Correlation between ELABELA or Apelin and study variables

We further analyzed the correlation between ELABELA or Apelin and study variables in all subjects (Table 4). BNP levels (r = − 0.704, p < 0.001), creatine levels (r = − 0.235, p < 0.001), urea nitrogen levels (r = − 0.284, p < 0.001), uric acid levels (r = − 0.110, p = 0.045), LAD (r = -0.265, p < 0.001), LVEDd (r = − 0.231, p < 0.001), LVEDs (r = − 0.223, p < 0.001), LVPW (r = − 0.167, p = 0.002), NYHA function grade (r = − 0.700, p < 0.001) were negatively related to plasma ELABELA levels. In contrast, diastolic blood pressure (r = 0.164, p = 0.003), mean arterial pressure (r = 0.117, p = 0.033), LDL-c levels (r = 0.134, p = 0.014), HDL-c levels (r = 0.126, p = 0.021), total cholesterol levels (r = 0.207, p < 0.001), total cholesterol levels (r = 0.207, p < 0.001), triglyceride levels (r = 0.164, p = 0.003) and LVEF (r = 0.183, p = 0.001) positively correlated to plasma ELABELA levels (Table 4).

**Table 4:** Spearman correlation between ELABELA and Apelin and study variables in all subjects.

Parameters	Plasma ELABELA, ng/ml		Plasma Apelin, pg/ml	
	*r* value	*P* value	*r* value	*P* value
Age, years	0.427	0.574	0.340	0.436
Sex	0.081	0.138	0.024	0.666
Body mass index, kg/m^2^	0.254	0.082	0.184	0.079
Coronary artery disease	0.004	0.949	0.175	0.001**
Diabetes mellitus	− 0.098	0.072	− 0.020	0.717
Chronic renal failure	0.052	0.264	0.044	0.687
Hypertension	0.083	0.130	− 0.030	0.581
Systolic blood pressure, mmHg	0.038	0.488	− 0.076	0.166
Diastolic blood pressure, mmHg	0.164	0.003**	− 0.107	0.050
Mean arterial pressure, mmHg	0.117	0.033*	− 0.099	0.069
Heart rate, bpm	− 0.081	0.139	0.145	0.008**
Plasma BNP, pg/ml	− 0.704	< 0.001***	0.336	< 0.001***
Plasma creatine, μmol/l	− 0.235	< 0.001***	0.034	0.539
Plasma urea nitrogen, μmol/l	− 0.284	< 0.001***	0.138	0.011*
Plasma uric acid, μmol/l	− 0.110	0.045*	− 0.012	0.821
Plasma LDL-c, mmol/l	0.134	0.014*	− 0.094	0.087
Plasma HDL-c, mmol/l	0.126	0.021*	− 0.017	0.759
Plasma total cholesterol, mmol/l	0.207	< 0.001***	− 0.080	0.145
Plasma triglyceride, mmol/l	0.164	0.003**	− 0.087	0.113
Plasma ELABELA, ng/ml	–	–	− 0.229	< 0.001***
Plasma Apelin, pg/ml	− 0.229	< 0.001***	–	–
LVEF, %	0.183	0.001**	− 0.144	0.037*
LAD, mm	− 0.265	< 0.001***	0.295	< 0.001***
LVEDd, mm	− 0.231	< 0.001***	0.213	< 0.001***
LVEDs, mm	− 0.223	< 0.001***	0.187	0.001**
IVST, mm	− 0.102	0.061	0.135	0.013*
LVPW, mm	− 0.167	0.002**	0.108	0.048*
RVIDd, mm	− 0.045	0.416	0.068	0.218
NYHA function grade	− 0.700	< 0.001***	0.365	< 0.001***

BNP, B-type natriuretic peptide; LDL-c, low-density lipoprotein cholesterol; HDL-c, high-density lipoprotein cholesterol; LAD, left atrial diameter; LVEDd, left ventricular end-diastolic diameter; LVEDs, left ventricular end-systolic diameter; LVEF, left ventricular ejection fraction; IVST, interventricular septum thickness; LVPW, left ventricular posterior wall thickness; RVIDd, right ventricular internal dimension diameter. HF, heart failure. *P < 0.05; **P < 0.01; ***P < 0.001.

Significant values are in bold.

In contrast, coronary artery disease (r = 0.175, p = 0.001), BNP levels (r = 0.336, p < 0.001), urea nitrogen levels (r = 0.138, p = 0.011), LAD (r = 0.295, p < 0.001), LVEDd (r = 0.213, p < 0.001), LVEDs (r = 0.187, p = 0.001), IVST (r = 0.135, p = 0.013), LVPW (r = 0.108, p = 0.048), NYHA function grade (r = 0.365, p < 0.001) were positively related to plasma Apelin levels, while LVEF (r = − 0.144, p = 0.037) negatively correlated to plasma Apelin levels (Table 4). Furthermore, plasma ELABELA levels were negatively correlated to plasma Apelin levels (r = − 0.229, *p* < 0.001).

Furthermore, multiple linear regression analyses in all the participants were performed to determine the relationship between plasma ELABELA levels and plasma Apelin levels and clinical characteristics associated with HF (Table 5). We found a significant association between plasma ELABELA levels and diastolic blood pressure (β = 0.096, t = 2.611, p = 0.009), plasma BNP levels (β = − 0.001, t = − 8.127, p < 0.001), and LAD (β = − 0.096, t = − 2.521, p = 0.012), while plasma Apelin levels were only associated with LAD (β = 5.775, t = 2.653, p = 0.008).

**Table 5:** Multivariate linear regression analysis of ELABELA and Apelin with clinical characteristics associated with heart failure.

Parameters	Plasma ELABELA, ng/ml			Plasma Apelin, pg/ml		
	*β*	*t*	*P* value	*β*	*t*	*P* value
Age, years	0.283	4.934	0.272	2.760	2.786	0.316
Sex	0.674	1.520	0.130	17.667	0.694	0.488
Body mass index, kg/m^2^	0.468	1.347	0.281	12.364	0.509	0.504
Diabetes mellitus	− 1.527	− 0.848	0.397	− 85.644	− 0.830	0.407
Hypertension	0.879	1.470	0.143	18.666	0.543	0.588
Coronary artery disease	0.114	0.224	0.823	40.444	1.386	0.167
Systolic blood pressure, mmHg	− 0.043	− 1.750	0.081	0.863	0.614	0.540
Diastolic blood pressure, mmHg	0.096	2.611	0.009**	− 2.771	− 1.305	0.193
Heart rate, bpm	− 0.001	− 0.022	0.983	− 0.310	− 0.194	0.847
Plasma BNP, pg/ml	− 0.001	− 8.127	< 0.001***	− 0.004	− 0.714	0.476
Plasma LDL-c, mmol/l	− 1.703	− 0.849	0.397	75.922	0.660	0.510
Plasma HDL-c, mmol/l	− 2.061	− 0.974	0.331	103.819	0.856	0.393
Plasma total cholesterol, mmol/l	2.201	1.085	0.279	− 87.725	− 0.753	0.452
Plasma triglyceride, mmol/l	− 0.450	− 0.478	0.633	45.818	0.850	0.396
LVEF, %	0.010	0.313	0.755	0.200	0.107	0.915
LAD, mm	− 0.096	− 2.521	0.012*	5.775	2.653	0.008**
Plasma Apelin, pg/ml	− 0.001	− 0.022	0.983	–	–	–
Plasma ELABELA, ng/ml	–	–	–	− 2.059	− 0.641	0.522

BNP, B-type natriuretic peptide; LDL-c, low-density lipoprotein cholesterol; HDL-c, high-density lipoprotein cholesterol; LAD, left atrial diameter; LVEF, left ventricular ejection fraction. *P < 0.05; **P < 0.01; ***P < 0.001.

Significant values are in bold.

### Baseline clinical characteristics of patients with different levels of ELABELA or Apelin

We further divided all the HF patients into two groups, the high-ELABELA-level group, and the low-ELABELA-level group, or the high-Apelin-level group and the low-Apelin-level group (Table 6). The low-ELABELA-level group exhibited more male patients (56.4 vs. 43.5%, p = 0.006), lower diastolic blood pressure (73.4 ± 9.6 vs. 77.8 ± 10.0 mmHg, p = 0.001), lower mean arterial pressure (91.2 ± 11.3 vs. 95.3 ± 11.3 mmHg, p = 0.009), higher BNP levels [2745.7 (127.0, 27,753.0) vs. 830.7 (96.2, 10,362.0) pg/ml, p < 0.001], lower creatine levels [67.2 (57.8,82.4) vs. 69.6 (56.2, 81.5) μmol/l, p = 0.001], higher urea nitrogen levels [9.4 (3.0, 47.9) vs. 7.0 (2.9, 69.2) μmol/l, p = 0.010], and lower ELABELA levels (4.7 ± 2.4 vs. 12.1 ± 2.9 ng/ml, p < 0.001) than those in the high-ELABELA-level group. Echocardiographic data indicated that the low-ELABELA-level group had lower LVEF (58.6 ± 9.8 vs. 62.4 ± 6.9%, p = 0.001) and longer LAD (38.1 ± 8.4 vs. 34.3 ± 5.1 mm, p < 0.001), LVEDd (48.6 ± 8.2 vs. 45.4 ± 4.7 mm, p < 0.001), LVEDs (33.2 ± 8.3 vs. 29.9 ± 4.0 mm, p < 0.001), and LVPW (9.8 ± 1.3 vs. 9.4 ± 1.3 mm, p < 0.001) than the high-ELABELA-level group, indicating the worse left ventricular systolic function and larger atrial and ventricular chambers in the low-ELABELA-level group. Moreover, the low-ELABELA-level group had fewer NYHA I patients (2.8 vs. 50.0%, p < 0.001) and more NYHA III (24.1 vs. 12.0%, p < 0.001) and NYHA IV (62.0 vs. 19.4%, p < 0.001) patients than the high-ELABELA-level group.

**Table 6:** The demographic and baseline characteristics of the HF patients with low level and high level of ELABELA or Apelin.

Parameters	Plasma ELABELA			Plasma Apelin		
	Low (n = 108)	High (n = 108)	*P* value	Low (n = 108)	High (n = 108)	*P* value
Age, years	77.2 ± 10.6	74.0 ± 11.6	0.243	75.6 ± 11.8	76.6 ± 10.8	0.468
Male	61/108 (56.4%)	47/108 (43.5%)	0.006**	56/108 (51.9%)	52/108 (48.2%)	0.528
Body mass index, kg/m^2^	25.5 ± 3.2	25.9 ± 3.3	0.352	25.7 + 2.4	25.8 + 3.1	0.641
Comorbidities						
Coronary artery disease	22/108 (20.4%)	26/108 (24.1%)	0.426	18/108 (16.7%)	30/108 (27.8%)	< 0.001**
Diabetes mellitus	3/108 (2.8%)	2/108 (1.9%)	0.389	3/108 (2.8%)	2/108 (1.9%)	0.389
Chronic renal failure	3/108 (2.8%)	0/108 (0.0%)	0.001**	3/108 (2.8%)	0/108 (0.0%)	0.001**
Hypertension	13/108 (12.0%)	25/108 (23.1%)	0.001**	20/108 (18.5%)	18/108 (16.7%)	0.607
Systolic blood pressure, mmHg	126.9 ± 16.5	130.3 ± 16.1	0.124	129.8 ± 15.4	127.4 ± 17.2	0.266
Diastolic blood pressure, mmHg	73.4 ± 9.6	77.8 ± 10.0	0.001**	76.8 ± 9.7	74.3 ± 10.1	0.058
Mean arterial pressure, mmHg	91.2 ± 11.3	95.3 ± 11.3	0.009**	94.5 ± 11.0	92.0 ± 11.9	0.103
Heart rate, bpm	77.8 ± 9.1	77.0 ± 7.6	0.464	75.6 ± 8.3	79.2 ± 9.0	0.002**
Laboratory data						
Plasma BNP, pg/ml	2745.7 (685.2, 3751.1)	830.7 (128.9, 567.2)	< 0.001***	1779.6 (153.7, 2160.2)	1796.8 (377.9, 2190.1)	0.965
Plasma creatine, μmol/l	87.2 (57.5,91.9)	69.6 (50.1, 75.9)	0.001**	84.8 (57.0, 80.4)	82.1 (57.8, 83.0)	0.281
Plasma urea nitrogen, μmol/l	9.4 (5.4, 10.1)	7.0 (4.6, 7.4)	0.010*	8.2 (5.0, 8.2)	8.1 (5.0, 8.6)	0.878
Plasma uric acid, μmol/l	364.9 (279.3, 425.8)	361.1 (298.3, 429.3)	0.802	370.0 (299, 446)	356.0 (279.3, 415.3)	0.365
Plasma LDL-c, mmol/l	2.3 ± 1.0	2.3 ± 0.9	0.669	2.3 ± 1.0	2.3 ± 0.9	0.604
Plasma HDL-c, mmol/l	1.3 ± 0.5	1.4 ± 0.4	0.213	1.3 ± 0.4	1.4 ± 0.5	0.541
Plasma total cholesterol, mmol/l	4.2 ± 1.2	4.3 ± 1.2	0.547	4.3 ± 1.2	4.2 ± 1.1	0.832
Plasma triglyceride, mmol/l	1.4 ± 0.9	1.3 ± 0.6	0.455	1.4 ± 0.8	1.2 ± 0.7	0.061
Plasma ELABELA, ng/ml	4.7 ± 2.4	12.1 ± 2.9	< 0.001***	9.2 ± 4.5	7.6 ± 4.5	0.010*
Plasma Apelin, pg/ml	170.6 ± 29.3	133.9 ± 26.8	0.317	26.7 ± 9.0	278.8 ± 36.2	< 0.001***
Echocardiographic data						
LVEF, %	58.6 ± 9.8	62.4 ± 6.9	0.001**	62.3 ± 6.5	58.8 ± 10.1	0.003**
LAD, mm	38.1 ± 8.4	34.3 ± 5.1	< 0.001***	34.4 ± 5.3	38.1 ± 8.3	< 0.001***
LVEDd, mm	48.6 ± 8.2	45.4 ± 4.7	< 0.001***	45.5 ± 5.0	48.5 ± 8.1	< 0.001***
LVEDs, mm	33.2 ± 8.3	29.9 ± 4.0	< 0.001***	30.0 ± 4.3	33.1 ± 8.2	< 0.001***
IVST, mm	10.0 ± 1.3	9.8 ± 0.9	0.198	9.7 ± 0.9	10.0 ± 1.3	0.045*
LVPW, mm	9.8 ± 1.3	9.4 ± 1.3	0.028*	9.4 ± 1.0	9.8 ± 1.5	0.006**
RVIDd, mm	20.9 ± 3.3	20.2 ± 2.0	0.094	20.6 ± 2.6	20.5 ± 2.9	0.769
NYHA function grade						
NYHA I	3/108 (2.8%)	54/108 (50.0%)	< 0.001***	40/108 (37.0%)	17/108 (15.7%)	< 0.001***
NYHA II	12/108 (11.1%)	20/108 (18.5%)	0.363	18/108 (16.7%)	14/108 (13.0%)	0.782
NYHA III	26/108 (24.1%)	13/108 (12.0%)	< 0.001***	13/108 (12.0%)	26/108 (24.1%)	< 0.001***
NYHA IV	67/108 (62.0%)	21/108 (19.4%)	< 0.001***	37/108 (34.3%)	51/108 (47.2%)	0.006**

BNP, B-type natriuretic peptide; LDL-c, low-density lipoprotein cholesterol; HDL-c, high-density lipoprotein cholesterol; LAD, left atrial diameter; LVEDd, left ventricular end-diastolic diameter; LVEDs, left ventricular end-systolic diameter; LVEF, left ventricular ejection fraction; IVST, interventricular septum thickness; LVPW, left ventricular posterior wall thickness; RVIDd, right ventricular internal dimension diameter. HF, heart failure. NYHA, New York Heart Association. *P < 0.05; **P < 0.01; ***P < 0.001.

Significant values are in bold.

In contrast, Low-Apelin-level group exhibited lower heart rate (75.6 ± 8.3 vs. 79.2 ± 9.0 bpm, p = 0.002), higher ELABELA levels (9.2 ± 4.5 vs 7.6 ± 4.5 ng/ml, p = 0.010), and lower Apelin levels (26.7 ± 9.0 vs. 278.8 ± 36.2 pg/ml, p < 0.001) than those in high-Apelin-level group. Echocardiographic data indicated that the low-Apelin-level group had higher LVEF (62.3 ± 6.5 vs. 58.8%, p = 0.003) and shorter LAD (34.4 ± 5.3 vs. 38.1 ± 8.3 mm, p < 0.001), LVEDd (45.5 ± 5.0 vs. 48.5 ± 8.1 mm, p < 0.001), LVEDs (30.0 ± 4.3 vs. 33.1 ± 8.2 mm, p < 0.001), IVST (9.7 ± 0.9 vs. 10.0 ± 1.3 mm, p = 0.045), and LVPW (9.4 ± 1.0 vs. 9.8 ± 1.5 mm, p = 0.006) than the high-Apelin-level group, indicating the worse left ventricular systolic function and larger atrial and ventricular chambers in the high-Apelin-level group. Moreover, the low-Apelin-level group had more NYHA I patients (37.0 vs. 15.7%, p < 0.001) and fewer NYHA III (12.0 vs. 24.1%, p < 0.001) and NYHA IV (34.3 vs. 47.2%, p = 0.006) patients than the high-Apelin-level group.

### Diagnostic value of ELABELA and Apelin level for HF

Using univariate logistic regression analysis and multivariate logistic regression analysis, we further investigated the clinical characteristics associated with HF, which may be the underlying risk factor for HF development (Table 7). In univariate analysis, plasma BNP levels (OR 1.023, 95% CI 1.004–1.025, *P* = 0.003), plasma Apelin levels (OR 1.005, 95% CI 1.002–1.009, *P* = 0.004), plasma ELABELA levels (OR 0.731, 95% CI 0.666–0.801, *P* < 0.001), and LAD (OR 1.088, 95% CI 1.011–1.172, *P* = 0.025) were closely associated with the occurrence of HF. In multivariate analysis, plasma BNP levels (OR 1.008, 95% CI 1.006–1.012, *P* = 0.002), plasma Apelin levels (OR 1.015, 95% CI 1.003–1.017, *P* = 0.001), plasma ELABELA levels (OR 0.722, 95% CI 0.658–0.791, *P* < 0.001), and LAD (OR 1.098, 95% CI 1.021–1.181, *P* = 0.012) were associated with the occurrence of HF. These results indicated that reduced ELABELA or increased Apelin levels may be an underlying risk factor for HF progression.

**Table 7:** Predictors of baseline characteristics in multivariate logistic regression analysis in patients with heart failure.

Parameters	Univariate analysis			Multivariate anaysis		
	OR	95% CI	*P* value	OR	95% CI	*P* value
Age, years	1.043	1.018–1.069	0.518			
Body mass index, kg/m^2^	1.024	0.913–1.118	0.406			
Sex	0.731	0.393–1.358	0.321			
Hypertension	1.281	0.553–2.968	0.563			
Coronary artery disease	1.081	0.525–2.223	0.833			
Systolic blood pressure, mmHg	1.013	0.979–1.049	0.461	1.016	0.980–1.053	0.395
Diastolic blood pressure, mmHg	0.987	0.939–1.038	0.619	0.985	0.935–1.038	0.572
Heart rate, bpm	1.024	0.983–1.067	0.254	1.020	0.978–1.064	0.359
BNP, pg/ml	1.023	1.004–1.025	0.003**	1.008	1.006–1.012	0.002**
Plasma LDL-c, mmol/l	0.589	0.042–8.191	0.694	0.855	0.076–9.681	0.900
Plasma HDL-c, mmol/l	0.652	0.038–11.191	0.768	0.938	0.066–13.292	0.962
Plasma total cholesterol, mmol/l	1.465	0.101–21.362	0.780	0.973	0.082–11.532	0.983
Plasma triglyceride, mmol/l	0.733	0.212–2.530	0.623	0.853	0.269–2.707	0.787
Plasma Apelin, pg/ml	1.005	1.002–1.009	0.004**	1.015	1.003–1.017	0.001**
Plasma ELABELA, ng/ml	0.731	0.666–0.801	< 0.001***	0.722	0.658–0.791	< 0.001***
LVEF, %	0.974	0.915–1.037	0.412	0.958	0.901–1.019	0.177
LAD, mm	1.088	1.011–1.172	0.025*	1.098	1.021–1.181	0.012*

CI, confidence interval; OR, odds ratio; BNP, B-type natriuretic peptide; LDL-c, low-density lipoprotein cholesterol; HDL-c, high-density lipoprotein cholesterol. *P < 0.05; **P < 0.01;***P < 0.001.

Significant values are in bold.

**Figure 2:**
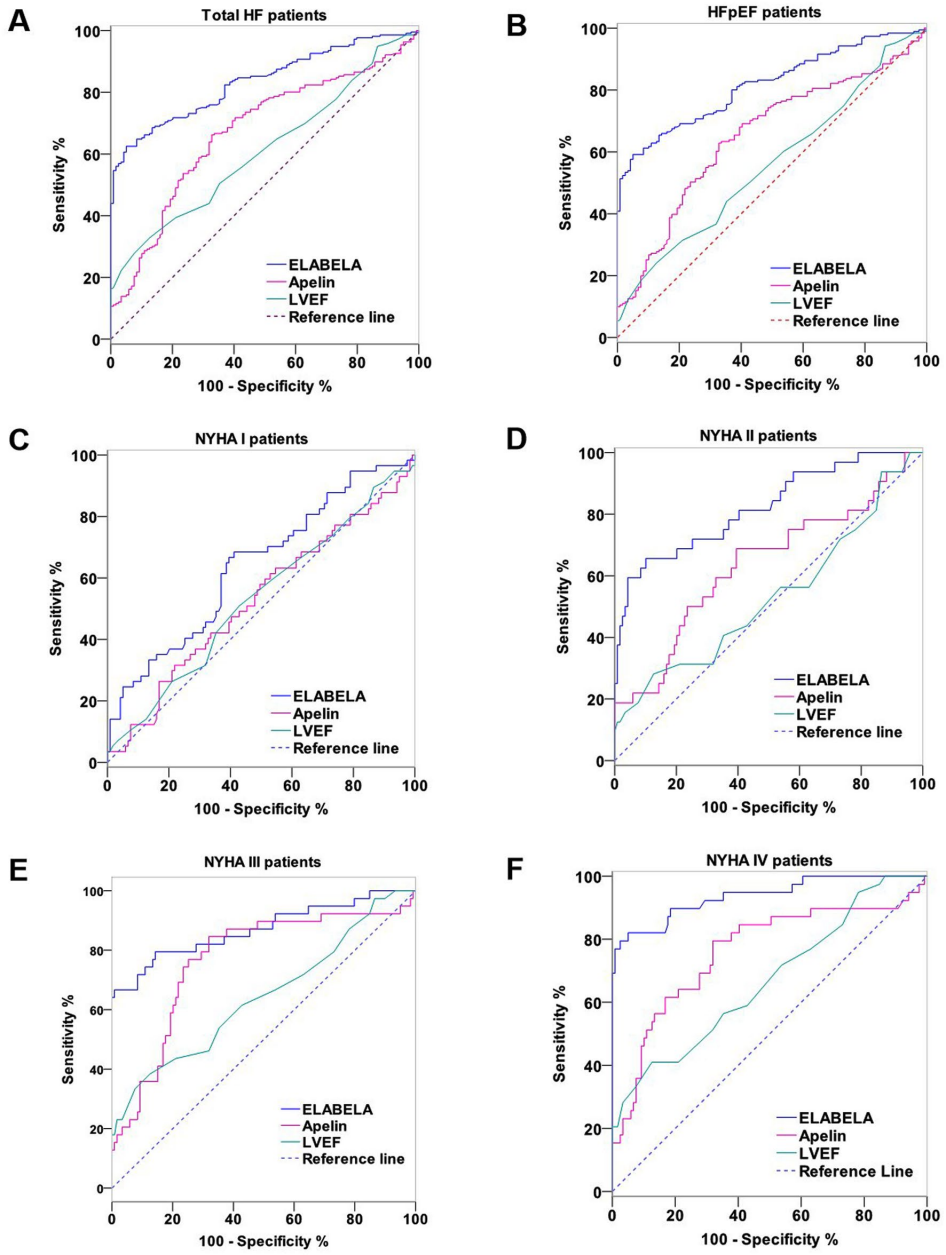
Receiver Operating Characteristic (ROC) curves of ELABELA, Apelin, and left ventricular ejection fraction (LVEF) levels for diagnostic value for heart failure.

To analyze the diagnostic value of ELABELA and Apelin, ROC curves were plotted for data for all Non-HF and HF patients (Table 8 and Fig. 2), and a pairwise comparison of ROC curves was performed by using the DeLong test (Table 9). For the total HF patients (Fig. 2A), the AUC area of ELABELA, Apelin, and LVEF was 0.835 ± 0.021 (95% CI 0.790–0.873), 0.673 ± 0.030 (95% CI 0.620–0.723), and 0.612 ± 0.031 (95% CI 0.557–0.664). The optimal cut-off point was 9.87 ng/ml (sensitivity 62.50%, specificity 94.96%), 36.75 pg/ml (sensitivity 66.20%, specificity 67.23%), and 59% (sensitivity 32.87%, specificity 87.39%), respectively. Using the DeLong test, there is a significant difference between the AUC values of ELABELA and that of both LVEF (∆AUC 0.223 ± 0.036, 95% CI 0.151–0.294, *P* < 0.001) and Apelin (∆AUC 0.162 ± 0.037, 95% CI 0.089–0.234, *P* < 0.001). For the HFpEF patients (Fig. 2B), the AUC area of ELABELA, Apelin, and LVEF was 0.817 ± 0.023 (95% CI 0.772–0.863), 0.653 ± 0.032 (95% CI 0.591–0.715), and 0.561 ± 0.033 (95% CI 0.497–0.626). The optimal cut-off point was 9.86 ng/ml (sensitivity 59.22%, specificity 95.82%), 36.81 pg/ml (sensitivity 62.84%, specificity 67.22%), and 59.52% (sensitivity 24.18%, specificity 87.40%), respectively. However, there is a significant difference between the AUC values of ELABELA and that of both LVEF (∆AUC 0.256 ± 0.040, 95% CI 0.177–0.335, P < 0.001) and Apelin (∆AUC 0.165 ± 0.039, 95% CI 0.088–0.242, P < 0.001) in the HFpEF patients. Thus, the diagnostic values of ELABELA were significantly higher than that of Apelin or LVEF, while there is no difference between the diagnostic values of Apelin and that of LVEF in the total or HFpEF patients.

**Table 8:** Diagnostic value of Plasma ELABEA, Apelin, and LVEF for heart failure.

	AUC (Sensitivity × 100-Specificity)			Optimal cutoff point	Sensitivity (%)	Specificity (%)
	AUC	*P* value	95% CI			
Total HF patients						
ELABELA	0.835 ± 0.021	< 0.001***	0.790–0.873	9.88 ng/ml	62.52	95.82
Apelin	0.673 ± 0.030	< 0.001***	0.620–0.723	36.81 pg/ml	66.20	67.23
LVEF	0.612 ± 0.031	< 0.001***	0.557–0.664	59.52%	32.97	87.49
HFpEF patients						
ELABELA	0.817 ± 0.023	< 0.001***	0.772–0.863	9.86 ng/ml	59.22	95.82
Apelin	0.653 ± 0.032	< 0.001***	0.591–0.715	36.81 pg/ml	62.84	67.22
LVEF	0.561 ± 0.033	0.070	0.497–0.626	59.52%	24.18	87.40
NYHA I patients						
ELABELA	0.639 ± 0.045	0.002**	0.563–0.709	12.72 ng/ml	68.42	58.82
Apelin	0.527 ± 0.048	0.570	0.451–0.603	65.66 pg/ml	31.68	78.25
LVEF	0.531 ± 0.047	0.517	0.454–0.606	63.69%	50.98	57.14
NYHA II patients						
ELABELA	0.822 ± 0.046	< 0.001***	0.751–0.879	10.11 ng/ml	65.62	89.92
Apelin	0.636 ± 0.060	0.024*	0.554–0.712	30.08 pg/ml	68.85	60.50
LVEF	0.531 ± 0.063	0.618	0.449–0.613	59.52%	28.12	87.49
NYHA III patients						
ELABELA	0.871 ± 0.040	< 0.001***	0.809–0.919	20.96 ng/ml	79.57	85.76
Apelin	0.766 ± 0.047	< 0.001***	0.693–0.830	39.29 pg/ml	84.62	68.17
LVEF	0.643 ± 0.055	0.010*	0.563–0.717	59.52%	38.56	87.49
NYHA IV patients						
ELABELA	0.950 ± 0.017	< 0.001***	0.911–0.975	9.88 ng/ml	82.19	95.48
Apelin	0.741 ± 0.036	< 0.001***	0.676–0.799	39.40 pg/ml	79.57	68.13
LVEF	0.680 ± 0.039	< 0.001***	0.612–0.743	59.52%	41.03	87.49

AUC, area under curve; CI, confidence interval; HF, heart failure; NYHA, New York Heart Association; LVEF, left ventricular ejection fraction. *P < 0.05; **P < 0.01; ***P < 0.001.

Significant values are in bold.

**Table 9:** Pairwise comparison of diagnostic value of plasma ELABEA, Apelin, and LVEF for heart failure using the DeLong test.

	ELABELA			Apelin		
	∆AUC	95% CI	*P* value	∆AUC	95% CI	*P* value
Total HF patients						
ELABELA	–	–	–	0.162 ± 0.037	0.089–0.234	< 0.001***
Apelin	0.162 ± 0.037	0.089–0.234	< 0.001***	–	–	–
LVEF	0.223 ± 0.036	0.151–0.294	< 0.001***	0.061 ± 0.044	-0.025–0.015	0.162
HFpEF patients						
ELABELA	–	–	–	0.165 ± 0.039	0.088–0.242	< 0.001***
Apelin	0.165 ± 0.039	0.088–0.242	< 0.001***	–	–	–
LVEF	0.256 ± 0.040	0.177–0.335	< 0.001***	0.092 ± 0.047	–0.000–0.184	0.051
NYHA I patients						
ELABELA	–	–	–	0.111 ± 0.067	-0.021–0.243	0.099
Apelin	0.111 ± 0.067	-0.021–0.243	0.099	–	–	–
LVEF	0.108 ± 0.064	− 0.017–0.233	0.090	0.003 ± 0.065	-0.124–0.131	0.958
NYHA II patients						
ELABELA	–	–	–	0.186 ± 0.082	0.025–0.347	0.024*
Apelin	0.186 ± 0.082	0.025–0.347	0.024*	–	–	–
LVEF	0.290 ± 0.078	0.138–0.442	< 0.001***	0.104 ± 0.086	-0.064–0.273	0.224
NYHA III patients						
ELABELA	–	–	–	0.105 ± 0.064	-0.021–0.231	0.103
Apelin	0.105 ± 0.064	-0.021–0.231	0.103	–	–	–
LVEF	0.228 ± 0.065	0.101–0.356	< 0.001***	0.124 ± 0.077	-0.027–0.274	0.108
NYHA IV patients						
ELABELA	–	–	–	0.209 ± 0.038	0.135–0.284	< 0.001***
Apelin	0.209 ± 0.038	0.135–0.284	< 0.001***	–	–	–
LVEF	0.270 ± 0.043	0.186–0.353	< 0.001**	0.061 ± 0.051	-0.040–0.161	0.237

∆AUC, the change of area under curve; CI, confidence interval; HF, heart failure; NYHA, New York Heart Association; LVEF, left ventricular ejection fraction. *P < 0.05; **P < 0.01; ***P < 0.001.

Significant values are in bold.

For the NYHA I patients (Fig. 2C), the AUC area of ELABELA, Apelin, and LVEF was 0.639 ± 0.045 (95% CI 0.563–0.709), 0.527 ± 0.048 (95% CI 0.451–0.603), and 0.531 ± 0.047 (95% CI 0.454–0.606). The optimal cut-off point was 12.72 ng/ml (sensitivity 68.42%, specificity 58.82%), 65.66 pg/ml (sensitivity 31.68%, specificity 78.25%), and 63.69% (sensitivity 50.98%, specificity 57.14%), respectively. However, there is no significant difference between the AUC values of ELABELA and that of both LVEF (∆AUC 0.108 ± 0.064, 95% CI − 0.017–0.233, *P* = 0.090) and Apelin (∆AUC 0.111 ± 0.067, 95% CI 0.021–0.243, *P* = 0.099) in the NYHA I patients. For the NYHA II patients (Fig. 2D), the AUC area of ELABELA, Apelin, and LVEF was 0.822 ± 0.046 (95% CI 0.751–0.879), 0.636 ± 0.060 (95% CI 0.554–0.712), and 0.531 ± 0.063 (95% CI 0.449–0.613), and the optimal cut-off point was 10.11 ng/ml (sensitivity 65.62%, specificity 89.92%), 30.08 pg/ml (sensitivity 68.85%, specificity 60.50%), and 59.52% (sensitivity 28.12%, specificity 87.49%), respectively. A significant difference was found between the AUC values of ELABELA and that of both LVEF (∆AUC 0.290 ± 0.078, 95% CI 0.138–0.442, P < 0.001) and Apelin (∆AUC 0.186 ± 0.082, 95% CI 0.025–0.347, *P* = 0.024) in the NYHA II patients. For the NYHA III patients (Fig. 2E), the AUC area of ELABELA, Apelin, and LVEF was 0.871 ± 0.040 (95% CI 0.809–0.919), 0.766 ± 0.047 (95% CI 0.693–0.830), and 0.643 ± 0.055 (95% CI 0.563–0.717), and the optimal cut-off point was 20.96 ng/ml (sensitivity 79.57%, specificity 85.76%), 39.29 pg/ml (sensitivity 84.62%, specificity 68.17%), and 59.52% (sensitivity 38.56%, specificity 87.49%), respectively. There is a significant difference between the AUC values of ELABELA and that of LVEF (∆AUC 0.228 ± 0.065, 95% CI 0.101–0.356, *P* = 0.001) but not Apelin (∆AUC 0.105 ± 0.064, 95% CI − 0.021–0.231, *P* = 0.103) in the NYHA III patients. For the NYHA IV patients (Fig. 2F), the AUC area of ELABELA, Apelin, and LVEF was 0.950 ± 0.017 (95% CI 0.911–0.975), 0.741 ± 0.036 (95% CI 0.676–0.799), and 0.680 ± 0.039 (95% CI 0.612–0.743). The optimal cut-off point of ELABELA, Apelin, and LVEF was 9.88 ng/ml (sensitivity 82.19%, specificity 95.48%), 39.40 pg/ml (sensitivity 79.57%, specificity 68.13%), and 59.52% (sensitivity 41.03%, specificity 87.49%), respectively. A significant difference was also detected between the AUC values of ELABELA and that of both LVEF (∆AUC 0.270 ± 0.043, 95% CI 0.186–0.353, P < 0.001) and Apelin (∆AUC 0.209 ± 0.038, 95% CI 0.135–0.284, *P* = 0.024) in the NYHA IV patients. In contrast with ELABELA, no significant difference was detected between the AUC values of Apelin and that of LVEF in HF patients regardless of the severity stratified by NYHA grade. Additionally, A significant difference was found between the AUC values of Apelin and that of ELABELA only in the NYHA II and NYHA IV patients. Thus, the diagnostic values of ELABELA, Apelin, and LVEF were associated with the classification of NYHA. In particular, the diagnostic values of ELABELA were increased with the severity stratified by NYHA grade and higher than that of Apelin and LVEF.

## Discussion

The present study aimed to investigate the potential diagnostic value of plasma ELABELA in HF patients compared to LVEF and Apelin. Our data have demonstrated that plasma ELABELA was significantly reduced and correlated with increasing NYHA grade or decreasing LVEF, utterly opposite to the changes in plasma BNP. On the contrary, plasma Apelin was significantly elevated but was not affected by the severity stratified by NYHA grade, the reduction of LVEF, or the sustained increase of plasma BNP in HF patients. We found that the levels of plasma ELABELA were negatively associated with LAD, LVEDd, LVEDs, LVPW, and plasma BNP and positively correlated with LVEF. In contrast, the correlation between plasma Apelin levels and these parameters was utterly opposite to that of plasma ELABELA. Moreover, although the differences in sensitivity between ELABELA and Apelin or LVEF for the diagnosis of HF depend on the specific NYHA grade, the diagnostic values and specificity of plasma ELABELA level for HF were higher than those of plasma Apelin and LVEF. Thus, reduced plasma ELABELA level may be a novel promising diagnostic indicator for HF patients. These findings may guide the use of ELABELA as a screening indicator for HF patients and call for further preclinical and clinical evaluation of the cardioprotective property of ELABELA in the setting of HF and the underlying mechanisms.

Although several studies have demonstrated an increase in plasma Apelin levels in NYHA II and III HF patients^27,42^, some other studies have reported a significant downregulation in plasma Apelin levels in HF^21,22,24,43^ and atrial fibrillation (AF)^44^ patients. Here we also found an elevated plasma Apelin level in HF patients. The reasons for these discrepancies are unclear but could be related to the difference in the study population and/or the testing methods (especially the differences in Apelin ELISA kits). Nevertheless, these studies implied a close correlation between plasma Apelin and the pathogenesis of HF. Indeed, Apelin exhibited a cardioprotective action in dilated cardiomyopathy^45^ and HF^15,46^ in animals. Due to the similar bioeffects with Apelin, ELABELA also exhibited similar cardiovascular-protective actions^18,19^. The current study demonstrated that plasma ELABELA levels were significantly reduced with the severity stratified by NYHA grade or decreasing LVEF in HF patients compared with non-HF patients. Although a previous study by Ma et al. has demonstrated the declined plasma ELABELA levels in hypertensive patients with HF^41^, preliminary indicating the status of plasma ELABELA in HF patients. However, they cannot rule out the interference from other complications, including hypertension and renal impairment. What's more, previous reports have already shown that plasma ELABELA concentrations were increased in patients with coronary heart diseases^31–33^ but reduced in patients with congenital heart disease^34^, renal impairment^38^, hypertension^37^, and AF^35^, indicating the independent impact of these complications on plasma ELABELA levels. As an extension, our study excluded the influence of the above complications and demonstrated the association of the declined plasma ELABELA levels with a high risk of HF progression. In this regard, patients with lower circulating ELABELA exhibited more severe cardiac dysfunction than those with higher circulating ELABELA. In addition, our study also showed that ELABELA was also positively related to, while Apelin had a negative correlation with (no significant statistical significance) plasma LDL-c, HDL-c, cholesterol, and triglyceride. These results indicated that ELABELA might be similar to Apelin in participating in metabolic regulation or metabolic related diseases such as atherosclerosis^47–49^. Recently, a small cohort study has shown that plasma ELABELA levels were negatively associated with carotid intima-media thickness in hypertensive patients, indicating the potential involvement of reduced ELABELA in the pathogenesis of hypertension-associated subclinical atherosclerosis^50^. It is important to note that atherosclerosis is considered to be the main cause of most cardiovascular diseases worldwide, with ischemic heart disease as its main clinical manifestation^51^. Thus, these pieces of evidence further support the potential importance of the declined circulating ELABELA in HF pathophysiology. However, the relationship between ELABELA and the progression of atherosclerosis needs to be further clarified.

We further compared plasma levels of ELABELA versus Apelin in different types of HF. Interestingly, plasma levels of ELABELA gradually decreased with increasing NYHA grade. In contrast, plasma Apelin levels were initially elevated in NYHA II patients compared to non-HF and NYHA I patients and then slightly decreased in NYHA III and IV patients compared to NYHA II patients with no statistical significance. Similarly, plasma ELABELA levels in the HF patients with HFpEF, HFrEF, or HFmrEF were sequentially decreased and lower than that in the non-HF patients. In contrary, The HF patients with HFpEF, HFrEF, or HFmrEF had higher plasma Apelin levels than non-HF patients. Interestingly, neither plasma Apelin levels between the HFpEF and HFmrEF or HFrEF group nor between HFmrEF and HFrEF group showed notable differences. These results indicated that decreased ELABELA level is more closely associated with impaired left ventricular systolic function than Apelin. There is no relationship between plasma Apelin levels and cardiac function. Notably, worsened heart function has been known as an independent risk factor for adverse events in HF patients^52^. Thus, the reduced plasma ELABELA levels rather than elevated plasma Apelin levels may be connected to adverse events in HF patients. Although studies have reported the positive inotropic effects and anti-myocardial fibrosis actions of ELABELA and Apelin^14,16,17,53^, ELABELA exhibited more effectively improving left ventricular filling in rats with cecal ligation puncture-induced sepsis^17^, achieve pronounced effects on cardiac contractions^54^, and reduce blood pressure and improve cardiorenal dysfunctions in spontaneously hypertensive rats^55^. The positive inotropic effect of Apelin was not matched to the elevated plasma Apelin levels in patients with HF. The reasons for this are not yet clear. However, we speculate that the increased plasma Apelin levels may be a compensation to the reduced plasma ELABELA levels and contribute to the alleviation of HF. This is supported by the observation that plasma Apelin levels were negatively correlated to plasma ELABELA levels in HF patients in our study.

HFpEF is recognized as a heterogeneous clinical syndrome and accounts for at least 50% of all HF patients^56^. However, the diagnosis of HFpEF is still challenging. In this regard, the European Society of Cardiology (ESC) has offered a complex definition of HFpEF which includes the symptoms and signs of HF, with evidence of structural and/or functional cardiac abnormalities and/or raised natriuretic peptides, and with an LVEF more than 50%^57^. Similarly, clinicians in clinical practice have adopted another definition of HFpEF which includes an LVEF more than 40%, an elevated N-terminal pro-BNP (NT-proBNP) level, and a structural cardiac abnormality on echocardiography^57^. Of note, approximately 20% of HFpEF patients with have normal natriuretic peptide levels^58^ and guidelines use the combination of LVEF ≥ 50% with functional abnormality assessment with tissue Doppler imaging to diagnose HFpEF^57^. Thus, the diagnosis of HFpEF is more difficult than that of HFmrEF or HFrEF and there is still no simpler definition specifying the use of a combination of imaging or natriuretic peptides in the diagnosis of HFpEF. In the present study, the great majority of the HF patients were diagnosed with HFpEF (88.43%), the percentages of the HFmrEF and HFrEF patients were only 6.94% and 4.62%, respectively. The diagnostic value and specificity of plasma ELABELA in patients with HFpEF were comparable with that in All HF patients and significantly higher than those of Apelin or LVEF. This may suggest the diagnostic potential of the decreased ELABELA level when combined with an elevated BNP level and LVEF ≥ 50% for HFpEF patients. Although it is clear that ventricular diastolic dysfunction plays a key role in HFpEF progression^59^, diastolic dysfunction is not synonymous with HFpEF but considered as a part of the normal again process^56^. In this case, the benchmarking to LVEF is not used for the diagnosis of HFpEF in a meaningful manner. The determination of the diagnosis value of plasma ELABELA on HFpEF may be compensate for the shortcomings of LVEF. In addition, multiple non-diastolic abnormalities including diabetes, obesity, chronic kidney disease, and hypertension are risk factors for HFpEF^56^. However, the occurrence of diabetes, obesity, chronic kidney disease, and hypertension was very low in the present cohort. This may be related to the specific subtypes of HFpEF. Indeed, a clinical phenotypic classification of HFpEF has already reported, mainly including (1) Vascular–related HFpEF; (2) Cardiomyopathy-related HFpEF; (3) Right heart- and pulmonary-related HFpEF; (4) Valvular- and rhythm-related HFpEF; and (5) Extracardiac disease-related HFpEF^60^. Along this line, our data may indicate that the HFpEF patients in the cohort mainly belong to cardiomyopathy-related HFpEF or valvular- and rhythm-related HFpEF. Briefly, patients with HFpEF in the present cohort may be mainly caused by valvular heart disease and old myocardial infarction.

The various bioeffects of ELABELA or Apelin display important roles in HF development. We found that plasma ELABELA levels rather than Apelin levels were positively correlated with diastolic blood pressure and negatively correlated with plasma creatine and urea nitrogen. Chronic kidney disease and hypertension are closely associated with HF and are known as independent risk factors for HF progression ^39,40,61^. Hypertension or chronic kidney disease interacts with HF, which jointly deteriorates the patient’s physical condition. Increasing animal studies have demonstrated that peripheral ELABELA administration exhibits an antagonistic actions on multiple cardiovascular-related diseases including hypertension^62,63^ and kidney injury^64,65^. Moreover, the levels of plasma ELABELA were remarkably lower and significantly negatively correlated with albuminuria, systolic and diastolic blood pressure in patients with type 2 diabetes^38^ or essential hypertension^37^. Therefore, reduced circulating ELABELA levels might cause the elevation of the incidence of HF progression through hypertension or renal dysfunction. Along this line, ELABELA may be a potential therapeutic target/drug for HF. This assumption may be supported by the evidence from multiple animal studies that have already demonstrated the protective actions of peripheral ELABELA application on cardiac injury including ischemia/reperfusion injury, oxidative stress injury, hypertensive injury, and myocardial infarction^18^. However, there is currently a lack of direct clinical evidence for the therapeutic efficacy of ELABELA for HF patients, which awaits future clinical evaluation.

NT-proBNP and BNP, known as diagnostic indicators of HF^66,67^, were associated with the severity and mortality of HF^68^ and important predictors for adverse events in HF patients^69^. Plasma ELABELA was significantly negatively associated with plasma BNP, while plasma Apelin was markedly positively related to plasma BNP in the present study. The negative relationship between ELABELA and BNP suggested that ELABELA protects against HF, possibly via its positive inotropic effect and attenuating cardiac remodeling. While the positive correlation between Apelin and BNP may be a compensatory outcome of reduced plasma ELABELA that enhances inotropic action and abolishes cardiac remodeling, thus exerting an anti-HF effect. Along this line, we found that the correlations between plasma ELABELA and cardiac function-related parameters (LVEF, LAD, LVEDd, LVEDs, IVST, LVPW, and RVIDd assessed by electrocardiography) were opposite to that between plasma Apelin and the above indexes. Unfortunately, both BNP and LVEF have poor predictive power for HF due to their low specificity^6,7^. Our results indicated that decreased ELABELA level might be a novel promising screening indicator for HF. In the present study, multivariate linear regression analysis revealed that plasma BNP only exhibited a significant negative impact on plasma ELABELA levels but not plasma Apelin levels. Thus, declined ELABELA plasma levels might be an underlying risk factor for HF progression and a potential predictor of a worse prognosis for HF. This concept can be supported by the multiple protective effects of ELABELA, including antihypertensive, cardioprotective, and renoprotective effects^18,19^. Similar to previous reports^27,41^, compared to ELABELA, the LVEF had a lower diagnostic value for HF with a lower predictive sensitivity and a comparable predictive specificity. Notably, although the predictive sensitivity of plasma ELABELA for the diagnosis of HF is comparable to that of plasma Apelin, plasma ELABELA had a higher diagnostic value and predictive specificity than plasma Apelin. Therefore, ELABELA might be superior to Apelin and LVEF for the diagnosis and prognosis of HF. Combined assessment of BNP and ELABELA may provide potential benefits for the diagnosis of HF.

The present study has several limitations. First, the current study population included only subjects from a single center. Thus, our findings may not be suitable for all ordinary populations due to the sample selection bias, including population and region bias. However, our findings are at least applicable to Chinese patients. Second, the sample size was small, which may reduce the reliability of the subgroup analysis. Third, the data was only dependent on the ELABELA/Apelin ELISA assay, the method is limited by the specificity of the antibody used in the ELISA kit. Fourth, patients in HF and non-HF groups were included based on typical signs, symptoms, and plasma BNP concentrations, with a lacking of a healthy control group or an HF group of different origins, such as patients with ischemic HF. It is still unclear whether there are differences in the levels of plasma ELABELA between healthy individuals and HF patients of matched age or HF of different origins. Lastly, follow-up studies were not conducted and outcome data are not available. The correlation between ELABELA and major outcomes including hospitalization, readmission, and all-cause mortality due to HF in our cohort is unclear. Therefore, future longitudinal multicenter clinical studies with a larger sample size and a healthy control cohort or an HF cohort of different origins are necessary to further verify the effectiveness of ELABELA in clinical diagnostic practice.

## Data Availability

The raw data supporting the conclusions of this article will be made available by Dr. Chuanming Xu without undue reservation.
